# Supplementary material for: Gluten-free food database: the nutritional quality and cost of packaged gluten-free foods
Source: PeerJ. 2015 Oct 22;3:e1337. doi: 10.7717/peerj.1337 (PMC4627916; doi:10.7717/peerj.1337)
Supplement: Table S1 [file peerj-03-1337-s001.docx]

Table 1: Food categories. Detailed list of included packaged food products

|  | **Included foods** |
| --- | --- |
| **Flour/Bake mix** | Flour, bake mix for cakes, bake mix for pizza, breadcrumbs |
| **Bread and bakery** | Rustic bread, whole-grain bread, toast, buns, ciabatta bread, raisin bread, Scone, baguette, lye brezel, rusk, Crispbread, Wraps |
| **Pasta and cereal-based** | Fusilli, Spaghetti, Penne, Lasagne sheets, Vermicelli, Tagliatelli, Cous Cous |
| **Cereals** | Granola (chocolate), Granola (nuts), Cornflakes |
| **Cookie and Cakes** | Shortbread, Neapolitan wafers, Cookie (chocolate), Mignon wafers (hazelnut), Marble cake, Ladyfinger, Cookie (whole-grain), Granola bar, Cookie (orange), Apple strudel, Muffin |
| **Snacks** | Cracker, Brezels, Grissini, Saltsticks, Wafers (plain) |
| **Convenience** | Pizza (salami), Pizza (margherita), Lasagne, Chicken Nuggets, Fish sticks, Soup (potato and leek), Soup (mushrooms), Wafer-cone (icecream filling), Pudding (semolina), Baked pastry case, Wafer (Oblate), Rice Drink (natural), Flaky pastry, Frozen Cake (almond, chocolate) |
