# Supplementary material for: Gluten-free food database: the nutritional quality and cost of packaged gluten-free foods
Source: PeerJ. 2015 Oct 22;3:e1337. doi: 10.7717/peerj.1337 (PMC4627916; doi:10.7717/peerj.1337)
Supplement: Supplemental Information 1 [file peerj-03-1337-s002.doc]

Gluten-free packaged foods (162)

Food item duplicates in sample (86)

Total analyzed packaged gluten-free foods (63)

Nutrient information incomplete; resp. not available in databasees

(13)

**Identification**

**Screening**

**Included**

| **Identified food** | **Number of exluded foods (n)** | **Reason for exclusion** |
| --- | --- | --- |
| Flour, Bake mix (cake), Breadcrumbs, Crispbread, Bread (Rustic, Buckwheat, Sunflower seed, Millet, Rice, Sourdough) Bun, Ciabatta, Lye pretzel, Fusilli, Spaghetti, Penne, Vermicelli, Tagliatelli, Granola (nuts, fruits and chocolate), Cornflakes, Cookies (shortbread, chocolate and orange), Neapolitan wafers, Granula bar, Muffin (vanilla and chocolate), Cracker, Saltsticks, Wafers (plain), Rice Drink (chocolate, vanilla, original) | 86 | Duplicates/Similar Products |
| [Almond](https://www.dict.cc/englisch-deutsch/almond.html) [Crescent](https://www.dict.cc/englisch-deutsch/crescent.html), [Fried](https://www.dict.cc/englisch-deutsch/fried.html) [Grated](https://www.dict.cc/englisch-deutsch/grated.html) [Potatoes](https://www.dict.cc/englisch-deutsch/potatoes.html) (mix), Muffin (vanilla), [Cheese](https://www.dict.cc/englisch-deutsch/cheese.html) [Savouries](https://www.dict.cc/englisch-deutsch/savouries.html), Fl[orentine](https://www.dict.cc/englisch-deutsch/Florentine.html) [Biscuit](https://www.dict.cc/englisch-deutsch/biscuit.html), [Chocolate-Covered](https://www.dict.cc/englisch-deutsch/chocolate-covered.html) [Pretzel](https://www.dict.cc/englisch-deutsch/pretzel.html), Potato Chips | 7 | No products containing gluten to compare |
| Buns | 6 | nutrient information in the database incomplete |
| Beer | 1 | ingredients of the product missing |
