# Supplementary material for: Gluten-free food database: the nutritional quality and cost of packaged gluten-free foods
Source: PeerJ. 2015 Oct 22;3:e1337. doi: 10.7717/peerj.1337 (PMC4627916; doi:10.7717/peerj.1337)
Supplement: Supplemental Information 2 — Notes. Precision estimates for energy, carbohydrate and protein. Theoretical nutrient content divided by nutrient content from the food label in % [file peerj-03-1337-s003.docx]

Table 3 Precision estimates for energy, carbohydrate and protein

| Nutrient | Precision estimate |
| --- | --- |
| Energy | - 2.3% ± 2.8% |
| Carbohydrate | + 0.5% ± 1.5% |
| Protein | + 6.4% ± 4.6% |

*Notes.* Precision estimates for energy, carbohydrate and protein. *Theoretical nutrient content divided by nutrient content from the food label in %*
